# Discovery of an Indolizine‐Based Compound with Potent Aβ42 Disaggregation Activity

**DOI:** 10.1002/alz70861_108415

**Published:** 2025-12-23

**Authors:** Subin KWAK, Dageum Kang, Suhyun Ye, Soljee Yoon, Ikyon Kim, YoungSoo Kim

**Affiliations:** ^1^ Yonsei University, Incheon, Incheon Korea, Republic of (South); ^2^ Yonsei University, Yeonsu‐gu, Incheon Korea, Republic of (South)

## Abstract

**Background:**

Alzheimer’s disease (AD) is a progressive neurodegenerative disorder characterized by the pathological accumulation of amyloid‐beta (Aβ) plaques and neurofibrillary tangles, with Aβ42 aggregates playing a particularly neurotoxic and central role in disease progression. Disassembling these aggregates represents a promising therapeutic strategy for AD. In this study, we screened a library of 13 indolizine‐based derivatives, YIAD library, to identify compounds capable of disrupting pre‐formed Aβ aggregates.

**Method:**

A set of YIAD compounds was screened to identify small molecules capable of disrupting pre‐formed Aβ aggregates. Initial screening via Thioflavin T (ThT) fluorescence inhibition assay identified five compounds exhibiting significant anti‐aggregation effects. These candidates were further assessed for their disaggregation activity using ThT‐based fluorescence dissociation assays. This was further validated through plate‐based quantification assays, confirming its efficacy in dissociating β‐sheet‐rich Aβ42 fibrils. Mechanistic insights were obtained through peptide mapping, which revealed specific interaction sites on Aβ42, and a dissociation profiling analysis supported the strong activity of compounds. To evaluate the central nervous system (CNS) permeability of the test compound, the parallel artificial membrane permeability assay (PAMPA) was conducted.

**Result:**

Among the candidates, YIAD‐0708 demonstrated the most potent disaggregation activity. Additional validation using plate‐based quantification assays confirmed its ability to dissociate β‐sheet‐rich Aβ42 aggregates. Furthermore, Peptide mapping and dissociation profiling further supported the strong disaggregating activity of YIAD‐0708. Importantly, an in vitro assay confirmed that YIAD‐0708 is capable of efficiently crossing the blood‐brain barrier (BBB), suggesting its viability as a CNS‐active agent.

**Conclusion:**

These findings identify YIAD‐0708 as a promising lead compound with potent Aβ42 disaggregation activity and potential for CNS penetration. Future in vivo studies are planned to evaluate its therapeutic efficacy in AD models.

